# Inhalation in Consumption

**Published:** 1854-12

**Authors:** 


### INHALATION IN CONSUMPTION.

By means of extensive advertisements and certificates in the newspapers, public attention has been drawn to the subject. I should, perhaps, not have noticed it in these pages, but for the request of some of my subscribers. For some fifty years past, the treatment of affections of the lungs, by inhalation, has appeared, disappeared and reappeared, with locust-like regularity, but always with variations; a new substance being introduced at each new advent, so as to make it take. Warm water, vinegar, oxygen, the effluvia of a cow-house, iodine, turpentine, alcohol, and many other substances, all have their day, and die, the patient as well as the treatment. If any method of inhalation was even of frequent success, if it cured one case in twenty, of actual consumption, no newspaper advertisement or certificate would be needed, and in three months the successful man's office would be besieged night and day, and no hundred men could despatch the business. The very fact that it is necessary to follow up the advertising system in the papers issued on Saturdays and Sundays, the latter being unfortunately the day when the newspaper is read over and over three or four times, advertisements and all, is conclusive evidence that the success of medicated inhalations is not sufficiently striking to keep its head above water without outside aid and influences. The best thing to be inhaled, either in consumption or bronchitis, in any and every stage, is pure out-door air, which costs nothing, and is not hard to take, being at the same time the natural food of the lungs, the natural purifier of the blood, and having been fashioned by the Almighty for the accomplishment of that object, one would think it was the best, and that there could be no substitute.

### HOW TO AVOID CONSUMPTION.

Eat a great deal, and exercise a great deal in the open air to convert what you eat into pure, healthful blood. Do not be afraid of out-door air, day or night. Do not be afraid of sudden changes of weather; let no change, hot or cold, keep you in doors. If it is rainy weather, the more need for your going out, because you eat as much on a rainy day as on a clear day, and if you exercise less, that much more remains in the system of what ought to be thrown off by exercise, and some ill result, some consequent symptom, or ill feeling, is the certain issue. If it is cold out of doors, do not muffle your eyes, mouth and nose in furs, veils, woolen comforters, and the like; nature has supplied you with the best muffler, with the best inhaling regulator, that is, two lips; shut them before you step out of a warm room into the cold air, and keep them shut until you have walked briskly a few rods and quickened the circulation a little; walk fast enough to keep off a feeling of chilliness, and taking cold will be impossible. What are the facts of the case; look at railroad conductors, going out of a hot air into the piercing cold of winter and in again every five or ten minutes, and yet they do not take cold oftener than others; you will scarcely find a consumptive man in a thousand of them. It is wonderful how afraid consumptive people are of fresh air, the very thing that would cure them, the only obstacle to a cure being that they do not get enough of it; and yet what infinite pains they take to avoid breathing it, especially if it is cold; when it is known that the colder the air is the purer it must be, yet if people cannot get to a hot climate, they will make an artificial one, and imprison themselves for a whole winter in a warm room, with a temperature not varying ten degrees in six months; all such people die, and yet we follow in their footsteps. If I were seriously ill of consumption, I would live out of doors day and night, except it was raining or mid-winter, then I would sleep in an unplastered log house. My consumptive friend, you want air, not physic, you want pure air, not medicated air, you want nutrition, such as plenty of meat and bread will give ☞ and they alone; physic has no nutriment, gaspings for air cannot cure you; monkey capers in a gymnasium cannot cure you, and stimulants cannot cure you. If you want to get well, go in for beef and out-door air, and do not be deluded into the grave by newspaper advertisements, and unfindable certifiers.

---

This number closes our first year's labors. We began without a single subscription, our receipts have more than paid our expenses, and we have not a delinquent subscriber. We have made a good array of pleasant acquaintances, from many of whom we have received, unasked, material and moral countenance and encouragement, ending with a verbal and I believe a sincere "God speed you."

We think it most independent and best not to send the first number of the next year unordered to any present subscriber in or out of New York, unless to those with whom we are personally acquainted. If any one desires to take the journal, it is easy to hand a dollar to the Postmaster, who will order it, and if they do not desire it, it is easier to do nothing.

An Editor naturally feels that he has a claim on his subscribers, and certainly has a kindly attachment for them; I do and would drop the acquaintance of any one of mine regretfully. I trust that all will subscribe again, and that each one will send an extra dollar for a year's journal for their minister.

Four numbers will be sent for three dollars. For three dollars, two Journals and any two dollar weekly newspaper in New York will be sent for one year. The Journal and Harper's, or Putnam's Magazine for one year will be sent for three dollars, which is the annual subscription for either. If any present subscriber desires to drop the Journal, I recommend such to take the Scientific American of this city, or The Country Gentleman of Albany, as having more frequently valuable items in reference to health than any others, and what is more, such items are reliable.

